# Administration of Protein Hydrolysates from Anchovy (*Engraulis Encrasicolus*) Waste for Twelve Weeks Decreases Metabolic Dysfunction-Associated Fatty Liver Disease Severity in ApoE^–/–^Mice

**DOI:** 10.3390/ani10122303

**Published:** 2020-12-04

**Authors:** Jessica M. Abbate, Francesco Macrì, Fabiano Capparucci, Carmelo Iaria, Giovanni Briguglio, Luca Cicero, Andrea Salvo, Francesca Arfuso, Antonio Ieni, Giuseppe Piccione, Giovanni Lanteri

**Affiliations:** 1 Department of Veterinary Science, University of Messina, 98168 Messina, Italy; jabbate@unime.it (J.M.A.); fmacri@unime.it (F.M.); farfuso@unime.it (F.A.); giuseppe.piccione@unime.it (G.P.); glanteri@unime.it (G.L.); 2 Department of Chemical, Biological, Pharmaceutical and Environmental Sciences, University of Messina, 98166 Messina, Italy; ciaria@unime.it; 3 Veterinary Practitioner, 98168 Messina, Italy; giobri9@gmail.com; 4 Zooprophylactic Institute of Sicily “A. Mirri” (IZS), 90129 Palermo, Italy; cicero.luca@libero.it; 5 Department of Pharmaceutical Chemistry and Technologies, La Sapienza, University of Roma, 00185 Roma, Italy; andrea.salvo@uniroma1.it; 6 Department of Human Pathology of Adult and Evolutive Age “Gaetano Barresi”, Section of Pathology, University of Messina, 98125 Messina, Italy; antonio.ieni@unime.it

**Keywords:** hydrolysates, anchovy, MAFLD, ApoE-deficient mice

## Abstract

**Simple Summary:**

Metabolic dysfunction-associated fatty liver disease (MAFLD) is an increasing concern worldwide. It currently represents the main cause of chronic liver disease in humans in Western countries. Nutritional strategies based on fish-rich diets are considered helpful in the prevention of MAFLD, and are also thought to be beneficial for human health. In particular, cholesterol- and triacylglycerol-lowering effects are associated with fish-derived proteins or hydrolysates. Our findings suggest that supplementing the diet with 10% (*w*/*w*) anchovy protein hydrolysates has an anti-obesity effect together with an improvement in lipid metabolism and a reduction in liver fat content and high-fat diet-induced liver disease. By virtue of their nutritional value and functional proprieties, anchovy by-product protein hydrolysates may be an efficient nutritional strategy in MAFLD prevention and treatment.

**Abstract:**

Metabolic dysfunction-associated fatty liver disease (MAFLD) includes several diseases, ranging from simple steatosis to steatohepatitis, fibrosis and cirrhosis. Fish-rich diets are considered helpful in the prevention of MAFLD, and the enzymatic hydrolysis of fish waste has been explored as a means of obtaining high-value protein hydrolysates, which have been proven to exert beneficial bioactivities including anti-obesity and hypocholesterol effects. This study aimed to assess the effect of the administration of protein hydrolysates from anchovy waste (APH) for 12 weeks on attenuated high-fat diet-induced MAFLD in apolipoprotein E-knockout mice (ApoE^–/–^). Thirty ApoE^–/–^ mice were divided into two groups (*n* = 15/group) and fed a high-fat diet (HFD), with and without the addition of 10% (*w*/*w*) APH. After 12 weeks, serum and hepatic lipid profiles, hepatic enzyme activities, liver histology and immunohistochemistry were analyzed to assess hepatic steatosis, inflammation and fibrosis. Twelve-weeks on a 10% (*w*/*w*) APH diet reduces total cholesterol and triglyceride serum levels, hepatic enzyme activity and hepatic triacylglycerol content (*p* < 0.0001), and results in a reduction in hepatic fat accumulation and macrophage recruitment (*p* < 0.0001). The results suggest that a 10% APH diet has an anti-obesity effect, with an improvement in lipid metabolism, hepatic steatosis and liver injury as a result of a high-fat diet. Protein hydrolysates from fish waste may represent an efficient nutritional strategy in several diseases, and their use as nutraceuticals is worthy of future investigation.

## 1. Introduction

Metabolic dysfunction-associated fatty liver disease (MAFLD), formerly named non-alcoholic fatty liver disease (NAFLD), is the most common cause of chronic liver disease in Western countries and is described as a growing public health problem, globally [1,2,3,4]. MAFLD includes several potentially progressive pathological features that range from hepatic steatosis to steatohepatitis, to the onset of fibrosis, cirrhosis and hepatocellular carcinoma [1,5,6]. Fatty liver is a simple hepatic steatosis characterized by excessive triacylglycerol accumulation (>55 mg triglycerides/g liver) [7] and micro/macro lipid vesicular formation in hepatocytes. In 30–40% of patients, hepatic steatosis evolves to steatohepatitis, which is characterized by steatosis with hepatocyte injury (ballooning), inflammation, and increased risk for liver fibrosis, cirrhosis and/or carcinogenesis [5,6]. Steatohepatitis is also associated with increased risk of cardiovascular adverse events, which represent the main MAFLD-related cause of death [8]. MAFLD is recognized as the hepatic manifestation of metabolic syndrome, and over-nutrition with visceral adiposity, diabetes, insulin resistance and dyslipidaemia are well-known related risk factors [5,9,10]. Nevertheless, the pathogenic mechanisms involved in disease progression are still poorly understood because they involve a complex interplay between genetic predisposition and risk factors [5,10,11,12,13]. Diet-induced mouse models that mimic human pathology have been employed to reproduce MAFLD, to improve current knowledge regarding pathogenesis and to find suitable therapeutic strategies [14,15]. In particular, apolipoprotein E-knockout (ApoE^–/–^) mice represent a valid animal model suitable for the assessment of liver disease [14,15]. Apolipoprotein E (Apo E) is a multifunctional protein that is mainly involved in the maintenance of plasma lipid homeostasis, which participates in the distribution/redistribution of lipids [16]. As a consequence, deficiency in ApoE rapidly induces dyslipidaemia and hypercholesterolemia in ApoE^–/–^ mice, leading to hepatic steatosis and liver injury [17,18,19]. Despite the increasing global incidence of MAFLD in the human population, there are currently no approved pharmacological therapies, and lifestyle intervention strategies are considered key factors in preventing liver diseases [9]. Diets with high-fat content, especially high saturated fatty acids, promote the onset of hepatic steatosis and progression to steatohepatitis [20,21,22]. Interestingly, the Mediterranean diet, which is composed of high monounsaturated fatty acids and a balanced omega-6 to omega-3 fatty acid ratio composition, provides a lower risk of MAFLD [23,24]. Fish-rich diets are considered helpful in the prevention and treatment of MAFLD due to the high amount of n-3 polyunsaturated fatty acids (PUFAs) in fish oil [25]. It has been well established that dietary intake of PUFAs has a triacylglycerol (TAG)-lowering effect, thus improving dyslipidaemia, hepatic steatosis and liver injury in MAFLD patients [26,27]. Additionally, fish proteins have been associated with a cholesterol-lowering effect and antioxidant proprieties [28,29,30,31]. Fish by-products, including skin, head, bone, viscera and frame, contain a high percentage of total fish proteins that may be useful a protein source for human consumption [32]. Several technologies have been used to obtain high-value products with remarkable biological and nutritional proprieties from fish wastes, and also because fish processing waste represents a serious problem of environmental and economic concern. Enzymatic hydrolysis of fish processing waste has been demonstrated to be an efficient strategy to obtain biologically active hydrolysates with high nutritional value, such as fish protein hydrolysates are characterized by a balanced amino acids composition and bioactive peptides [28,32,33,34]. Additionally, protein hydrolysates from fish by-products have been proven to promote various beneficial biological activities, including immunomodulatory, anti-inflammatory, hypocholesterolemic, antidiabetic, antihypertensive, antiatherogenic and antioxidative effects [35,36,37,38] and their potential application in the prevention and treatment of human diseases is worthy of investigation.

Therefore, the main goal of this study was to assess the effect of the administration of protein hydrolysates from anchovy (*Engraulis encrasicolus*) waste for 12 weeks on attenuating high-fat diet-induced MAFLD in ApoE^–/–^ mice. The results will improve current knowledge of the biological and nutritional value of protein hydrolysates from fish waste and MAFLD so as to enhance the use of protein-rich fish waste in human health, and also reduce fish-industry derived pollution.

## 2. Materials and Methods

### 2.1. Animals and Experimental Design

Thirty 6-month old female B6.129P2-ApoE^–/–^ mice (C57BL/6 genetic background) were used in the study. Animals were allocated to sterile filter-top cages (5 mice/cage), kept in standard laboratory conditions (21 ± 1 °C, 55 ± 5% humidity, with a 12 h light/dark cycle) and received water and chow *ad libitum* before the beginning of the experimental study. After 1-week acclimatization, mice were randomly distributed into two different groups (*n* = 15/group) according to the diet type. Mice in both groups were fed a high-fat diet (HFD) containing 21% fat (>60% saturated fatty acids), 19.5% casein, 0.21% cholesterol, 34% sucrose (TD.88137 Envigo; Indianapolis, IN, USA) for twelve weeks; however, for the experimental group, 10% of casein was replaced with the same amount of protein hydrolysates from anchovy waste (*w*/*w*) (APH). Mice enrolled in both groups received water and chow during the experimental study. At inclusion (study day 0; T0), the body weight of the mice was determined (control group mean 15.83 ± 0.47 g; range 15.00–16.30 g; experimental group: mean 16.00 ± 0.22 g; range 15.70–16.30 g), and during the study period, mice were checked daily for health status, monitored for food intake and weighed weekly. After 12 weeks (study day 84; T84) the mice were sacrificed with an overdose of anesthetic (isoflurane >5%), and blood was sampled from a peripheral vein (lateral tail vein) and stored in a 2.5 mL cloth activator tube. Total cholesterol (TC; mg/dL), triglycerides (TG; mg/dL), aspartate amino transferase (AST; UI/L), and alanine aminotransferase (ALT; UI/L) levels were measured on serum samples using an automated clinical chemistry analyzer (Konelab 60I; Thermo Electron Corporation, Vantaa, Finland) and by means of commercially available kits. Finally, animal carcasses underwent necropsy, and all organs were stored for future analysis. In particular, livers were weighed and sampled for subsequent investigations. The Animal Welfare Organization (OPBA) approved the study (approval no. 771/2018-PR; 04/10/2018). All experimental procedures were conducted according to Italian regulations on use of animals for experiments (D.L 26/2014), and to European regulations (2010/63/UE) for animal care. In vivo experimental procedures were performed at the Experimental Zooprophylactic Institute of Sicily “A. Mirri”, Palermo (cod 28875, Ministerial authorization 14/2015-UT). All post-mortem analyses were carried out at the unit of Veterinary Pathology, Department of Veterinary Science, University of Messina (Sicily, Italy).

### 2.2. Anchovy Protein Hydrolysates

Fish protein hydrolysates used in the current study were prepared by enzymatic hydrolysis of anchovy by-products (viscera), as detailed by Mangano et al. [39]. Briefly, fish viscera were mechanically ground and crushed, heated to 55 °C and mixed with distilled water (1:3; *w*/*v*). Enzymatic hydrolysis was produced adding 3% (E/S, *w*/*w*) of a combination of Promatex, Flavourzyme 500 MG and Alcalase 2.4 L (1.1,1.1,0.9, *w*/*w*; Novozymes China Inc.; Guangzhou, China) as commercial proteases, and the mixture was incubated in a 5 L bioreactor (BIOSTAT^®^ Sartorius, Italy) with controlled parameters (pH 7.5, 50 °C, 150 rpm). After 3 h of hydrolysis the mixture was heated to 90 °C for 15 min to inactivate the enzymes, filtered and centrifuged (8000 rpm; 4 °C) for 15 min. The collected supernatant was dehydrated in a Mini Spray Dryer B-290 (Buchi Italia s.r.l., Cornaredo, Italy), and dried powder was collected in a single cyclone air separator system and used to determine the protein content by the 938.08 AOAC methodology (AOAC, 1993). The amino acid composition of the protein hydrolysates was then determined by means of high-performance liquid chromatography [39].

### 2.3. Histopathological Examination

For the histopathological investigations, liver tissue samples were sectioned; some were frozen at −80 °C and others were fixed in 10% buffered formalin until further analysis. Formalin-fixed, paraffin-embedded liver sections (5 µm) were stained with hematoxylin-eosin (H&E) for examination of liver morphology and with Mallory’s trichrome (Bioptica, 04-020802, Milano, Italy) for evaluation of fibrosis. For detection of lipids in the liver, 5 µm sections of snap-frozen liver samples were prepared using a cryostat (Microm 505M, Ramsey, MN, USA) and stained with Oil Red O (Sigma-Aldrich, 04-2209-23, Milano, Italy). Histological staining was analyzed by a pathologist blinded to the diet type and according to a MAFLD histological scoring system proposed by Kleiner et al., which semi-quantitatively evaluated steatosis (0–3), inflammation (0–2), hepatocellular ballooning (0–2) and fibrosis (0–4) [40]. Oil Red O-stained liver sections were visualized using a Leica DM6B microscope (Leica Camera, Wetzlar, Germany) and Leica Application Suite X software. Images at ×40 magnification were acquired using a Leica DFC 7000 T and fat accumulation was quantified using the color cube-based method with ImageJ software (ImageJ 1.52q, Bethesda, MD, USA). Quantification (indicated as % of positive stained area) was assessed on three representative sections from *n* = 6 animals per group and performed blinded.

### 2.4. Immunohistochemistry

Immunohistochemical staining was performed on 10 µm paraffin-embedded sections, developed from an Avidin-biotin complex (BioSpa 20143, Milano, Italy) and revealed by DAB (Diaminobenzidine, Vector Laboratories, Inc. U.S. Headquarters, Burlingame, CA, USA). Monoclonal rat anti-F4/80 (Abcam, Cambridge, UK; Product Code: ab16911; dilution 1:100) antibody labelling a 125 kDa extracellular macrophage membrane molecule and highly restricted to mature macrophage subpopulations residing in tissue, and monoclonal rat anti-CD3 (Abcam, Cambridge, UK; Product Code: ab11089; dilution 1:250) antibody labelling a highly conserved epitope of the CD3 molecule expressed by T lymphocytes were used. Positive and negative controls were always performed as reported by Iaria et al. [41]. Immunohistochemical labeling was quantified using the color cube-based method with ImageJ software (ImageJ 1.52q, Bethesda, MD, USA) and reported as a % of the positive stained area. Quantification was assessed on three representative sections from *n* = 6 animals per group and performed blinded.

### 2.5. Nuclear Magnetic Resonance (NMR) Analysis

NMR spectroscopy was employed as a tool for determining hepatic free cholesterol, cholesteryl ester and triacylglycerol content. Briefly, 500 mg of liver was homogenized in 1.5 mL solution of distilled water, containing KCl (5 mmol/L), EDTA and CaCl_2_ (1 mmol/L), with 1% NaN_3_, and the solution was adjusted to pH 7.4. Thereafter, 1 mL of CDCl_3_ was added. After sonication (30 min) and centrifugation (4500 rpm for 10 min), the water-soluble fraction was separated from the lipophilic fraction in CDCl_3_. The hydrophilic fraction (500 µL) was deposited in a 5 mmol/L NMR test-tube, containing sodium 3-trimethylsilyl propionate (TPS; 10 mmol/L), 99.8% D_2_O, MnSO_4_ (0.6 mmol/L). NMR analysis was performed using an NMR Bruker Avance III 500 MHz spectrometer (Bruker, Milano, Italy) equipped with a SMARTprobe and managed by TOPSPIN 4.2 as a workstation. The NOESY-presat procedure was adopted to suppress the water signal, thus maintaining a good baseline profile. Pre-saturation time was set to 1.5 s and power was consequently optimized. Acquisition time was 3 s and a 5 s time delay was added to allow full relaxation; a spectral width of 5500 Hz (11 ppm) was set for each experiment. Chemical shifts were referenced to the CaEDTA sharp signal at 2.519 ppm whereas the TSP (0.31 ± 0.005 ppm) was used as a quantitative reference [42,43].

### 2.6. Data Analysis

Data are presented as mean ± standard deviation (SD) for *n* = 6 mice per group. All data were tested for normal distribution using the Kolmogorov–Smirnov test. Two-way ANOVA for repeated measures was applied to evaluate the effect of the different diet and of time on body weight (*p* < 0.001). Unpaired Student’s *t*-test was performed to assess statistically significant differences between groups in serum levels of lipids and hepatic enzymes, hepatic lipid content, histological and immunohistochemical findings. Statistical analyses were performed using GraphPad Prism version 7.00 (GraphPad Software, San Diego, CA, USA).

## 3. Results

### 3.1. Animals

**Figure 1:**
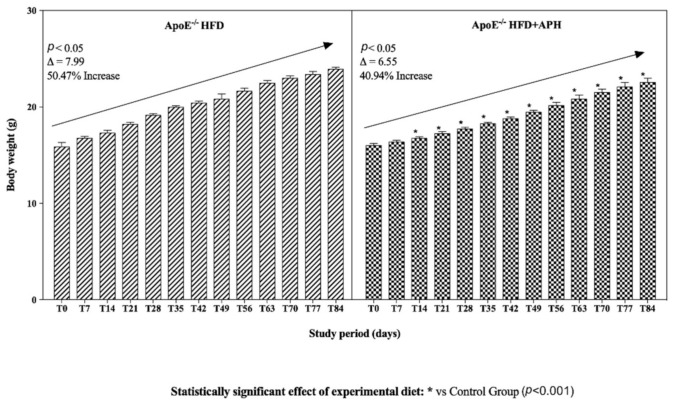
Body weight trends in mice throughout study period. An increasing trend in body weight was observed in ApoE^–/–^ mice of both groups (*p* < 0.05), with a percentage increase of 50.47% in ApoE^–/–^ mice fed HFD and 40.94% in ApoE^–/–^ fed HFD + APH. Body weight gain was significantly lower in ApoE^–/–^ fed HFD + APH from T14 to T84 compared to the control group (*p* < 0.001). Data are presented as mean ± SD for *n* = 6 mice per group. Abbreviations: HFD high-fat diet, APH anchovy protein hydrolysates.

Statistical analysis of the data showed that diet and time had a significant effect on the body weight values measured in the studied animals. An increasing trend in body weight was observed in both groups (*p* < 0.05), with a percentage increase of 50.47% in ApoE^–/–^ mice fed a HFD and an increase of 40.94% in mice fed a HFD + APH. Body weight gain was significantly lower in ApoE^–/–^ HFD + APH from T14 to T84 compared to the control group (*p* < 0.001) (Figure 1).

**Figure 2:**
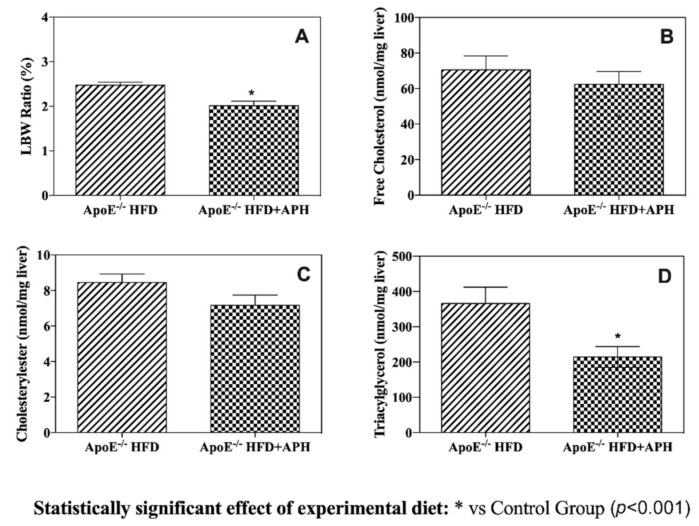
Liver-to-body weight ratio (LBW) (**A**) and levels of hepatic free cholesterol (**B**) cholesteryl ester (**C**) and triacylglycerol (**D**). LBW in ApoE^–/–^ mice fed with HFD was significantly increased (i.e., 2.47%) compared to ApoE^–/–^ mice fed with HFD + APH (i.e., 2.01%) (*p* < 0.0001). No statistical difference was found in either hepatic free cholesterol (**B**) or cholesteryl ester (**C**), whereas hepatic triacylglycerol (**D**) content was significantly reduced in ApoE^–/–^ mice fed HFD+APH (214.39 ± 29.67 nmol/mg) compared to values obtained from ApoE^–/–^ control mice fed HFD (365.97 ± 46.20 nmol/mg). Data are presented as mean ± SD for *n* = 6 mice per group. Abbreviations: HFD, high-fat diet, APH, anchovy protein hydrolysates.

Food consumption throughout the study ranged from 3.16 g/day to 6.04 g/day for individual mice, and there was no significant difference in food intake between the experimental and control groups. The liver-to-body weight ratio (LBW) of ApoE^–/–^ mice fed with HFD was 2.47%, whereas the LBW for mice in the experimental group was statistically lower (i.e., 2.01%) (*p* < 0.0001) (Figure 2).

**Figure 3:**
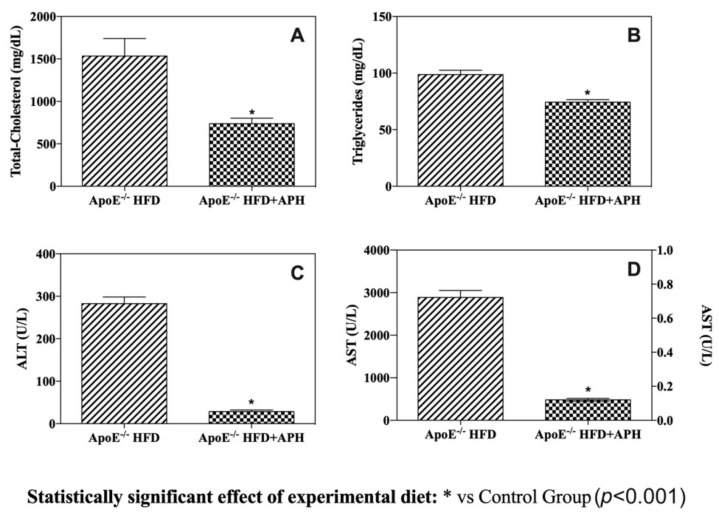
Serum lipid levels and hepatic enzyme activity. Administration of APH for 12 weeks produced significant serum total cholesterol and triglycerides-lowering effects (**A**,**B**). Serum ALT (alanine aminotransferase) (**C**) and AST (aspartate amino transferase) (**D**) levels were statistically lower in ApoE^–/–^ HFD + APH compared to the control group (*p* < 0.0001), suggesting minor damage of the hepatocytes. Data are presented as mean ± SD for *n* = 6 mice per group. Abbreviations: HFD high-fat diet, APH anchovy protein hydrolysates.

As shown in Figure 3, the serum values of TC and TG were lower in HFD+APH-fed ApoE^–/–^ mice (TC: 737.83 ± 64.74 mg/dL; TG: 74.33 ± 5.57 mg/dL) compared to the values obtained from HFD-fed ApoE^–/–^ mice (TC: 1534.33 ± 0.21 mg/dL; TG: 98.67 ± 9.67 mg/dL) (*p* < 0.001). The intake of a HFD for 12 weeks typically induced hyperlipidaemia in mice of both groups, although the addition of APH to the HFD significantly attenuates both total cholesterol and triglyceride serum levels. Also, serum ALT and AST levels were statistically higher in HFD-fed mice (ALT: 282.33 ± 15.88 U/L; AST: 2.88 ± 0.16 U/L) compared to HFD+APH mice (ALT: 28.33 ± 3.88 U/L; AST: 481.17 ± 33.59 U/L) (*p* < 0.0001) (Figure 3). In particular, a significant 10- and 6-fold increase in serum ALT and AST concentrations, respectively, was observed.

### 3.2. Macroscopic and Histological Evaluation

**Figure 4:**
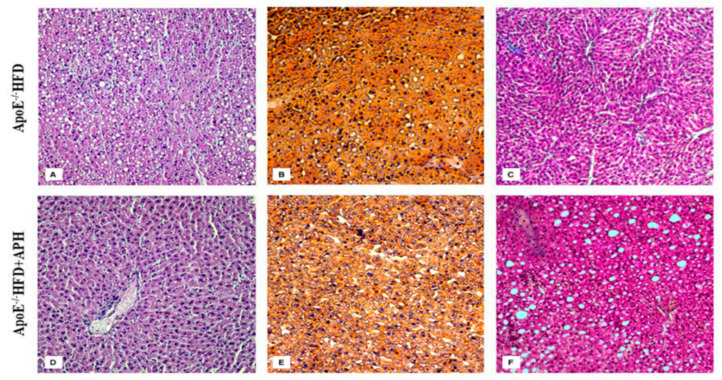
Hepatic histology. Representative liver sections of H&E (**A**,**D**) Oil Red O (**B**,**E**) and Mallory’s Trichrome (**C**,**F**) histological staining of ApoE^–/–^mice included in both groups (Scale bar, 100 µm). Abbreviations: HFD high-fat diet, APH anchovy protein hydrolysates.

Initial macroscopic investigation revealed that the livers of mice included in both groups were clearly increased in volume, with a smoother and clearer surface than normal, suggesting the presence of hepatic steatosis, although the macroscopic lesions were more evident in ApoE^–/–^HFD mice. H&E stained liver sections showed moderate macro-vesicular and micro-vesicular steatosis throughout the hepatic lobule with minimum hepatocellular ballooning degeneration (grade 1 ballooning), no inflammatory foci and no hepatic fibrosis in ApoE^–/–^HFD mice. Oil Red O stained sections showed the development of grade 2 steatosis (33–66%), whereas Mallory’s trichrome staining was negative. Histopathological evaluation in ApoE^–/–^HFD + APH-fed mice revealed grade 1 steatosis (5–33%), which was mainly represented by micro-vesicular steatosis with a significant attenuation of fatty liver alterations and hepatocyte injury. In the experimental group, the effects of HFD on hepatic steatosis were attenuated by APH intake (Figure 4).

**Figure 5:**
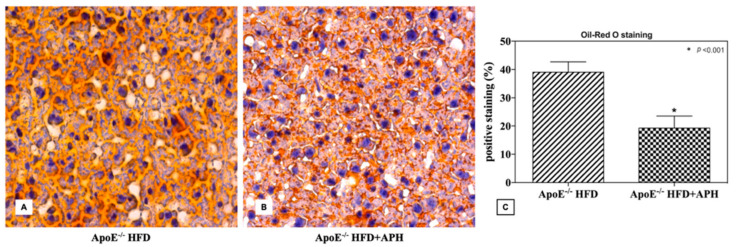
Representative liver sections (**A**,**B**) and quantification (% of positive stained area) of Oil-Red O histological staining (**C**). ApoE^–/–^mice fed a HFD showed a statistically twofold increase in hepatic fat accumulation (39.06 ± 3.67%) compared to ApoE^–/–^mice fed with a APH diet (19.26 ± 4.32%). Data are presented as mean ± SD for *n* = 6 mice per group (Scale bar, 50 µm). Abbreviations: HFD high-fat diet, APH anchovy protein hydrolysates.

A statistically significant two-fold increase in hepatic fat accumulation was assessed in Oil Red O staining in the ApoE^–/–^HFD group (39.06 ± 3.67%) compared to the ApoE^–/–^HFD+APH group (19.26 ± 4.32%) (*p* < 0.001) (Figure 5).

### 3.3. Immunohistochemical Evaluation

**Figure 6:**
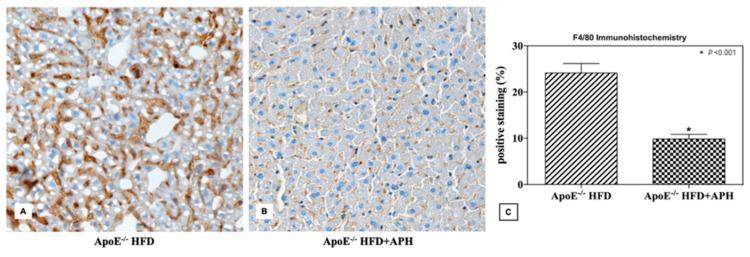
Representative liver sections (**A**,**B**) and quantification (% of positive stained area) of F4/80 immunohistochemistry (**C**). ApoE^–/–^mice fed a HFD showed a statistically significant threefold increase in F4/80 expression levels (24.14 ± 2.04%) compared to ApoE^–/–^ mice fed with a APH diet (9.81 ± 1.03%) (*p* < 0.001). Data are presented as mean ± SD for *n* = 6 mice per group (Scale bar, 50 µm). Abbreviations: HFD high-fat diet, APH anchovy protein hydrolysates.

Immunohistochemical investigations showed a significant increase in F4/80 positive staining in ApoE^-/-^ mice fed with HFD compared to mice fed with HFD + APH (*p* < 0.001), indicating an increase in the recruitment of macrophages in the liver of ApoE^–/–^HFD mice, whereas CD3 expression was negative in both ApoE^–/–^HFD and HFD + APH liver sections. An approximately three-fold increase in F4/80 expression levels was observed in HFD fed mice (24.14 ± 2.04%) compared to HFD + APH fed mice (9.81 ± 1.03%) (*p* < 0.001) (Figure 6).

### 3.4. Hepatic Lipid Content

APHs significantly (*p* < 0.001) reduced hepatic triacylglycerol content in ApoE^–/–^HFD + APH mice (214.39 ± 29.67 nmol/mg) compared to values obtained from the control ApoE^–/–^HFD (365.97 ± 46.20 nmol/mg) group, which suggests that fish protein hydrolysates have a triacylglycerol-lowering effect. On the contrary, statistical analysis of the data did not reveal significant differences in either hepatic free cholesterol (ApoE^–/–^HFD: 70.55 ± 7.83 nmol/mg; ApoE^–/–^HFD + APH: 62.42 ± 7.24 nmol/mg) or cholesteryl ester (ApoE^–/–^HFD: 8.45 ± 1.17 nmol/mg; ApoE^–/–^HFD + APH: 7.16 ± 1.43 nmol/mg) content between the two groups, although a difference in mean values was observed (Figure 2).

## 4. Discussion

Apolipoprotein E deficiency in ApoE^–/–^ mice seems to be strongly correlated with MAFLD development and plays an important role in metabolic syndrome [17,44,45], as the absence of ApoE spontaneously induces hypercholesterolemia, obesity and atherosclerosis [17,19]. The administration of a HFD in ApoE^–/–^mice enhances the effect of the knockout, which increases susceptibility to liver injury [45]. Although ApoE^–/–^mice are an experimental animal model used mainly in cardiovascular research, they have recently gained attention in an MAFLD study [14,15]. In the current study, the effect of 10% APH (*w*/*w*) on lipid metabolism and MAFLD development in ApoE^–/–^ mice was investigated. The obtained results demonstrate that APH consumption mitigates the effect of a HFD on hepatic lipid accumulation and hepatocytes injury, thus suggesting its potential employment in MAFLD treatment. In particular, although twelve weeks on a HFD led to a significant body weight gain in mice in both the ApoE^–/–^HFD and ApoE^–/–^HFD + APH groups, eating fish protein hydrolysates resulted in a slowdown in body weight gain in the experimental group, starting from study day 14 (T14) until the end of the study (T84). This corroborates the anti-obesity effect of APH already suggested in previous studies [35]. The liver-to-body weight ratio increased more in ApoE^–/–^HFD mice than in HFD+APH-fed mice, suggesting more marked hepatomegaly in the control group. Liver weight increases due to hepatic steatosis, which is characterized by lipid deposition in the cytoplasm and hepatocyte hypertrophy. Consistent modification of liver profiles, with an increase in volume and discoloration was observed in mice included in both groups. Histological assessment confirmed the development of moderate hepatic steatosis in ApoE^–/–^ HFD mice. Steatosis was diffuse, with macro- and micro-vesicular formation within the hepatocytes and cellular damage. Conversely, consuming fish hydrolysate for 12 weeks significantly reduced hepatic steatosis in ApoE^–/–^ HFD + APH-fed mice, and histological evaluation of liver sections revealed mild micro-vesicular steatosis and attenuation of hepatocyte injury. Quantification of Oil Red O staining confirmed a two-fold increase in hepatic fat accumulation in ApoE^–/–^HFD compared to APH-fed mice. Additionally, ApoE^–/–^ mice fed with HFD showed a significant three-fold increase in F4/80 positive staining, compared with fish hydrolysate-fed ApoE^–/–^mice, indicating greater recruitment of macrophages as a result of greater fat liver accumulation and cellular injury. Hepatocellular damage resulted in a marked increase in serum transaminases in ApoE^–/–^ HFD control mice. Moreover, a 10- and 6-fold increase in serum ALT and AST levels, respectively, was observed. ALT enzyme is a sensitive indicator of active liver damage, as it is abundantly present in the cytoplasm of hepatocytes and an increase in blood levels is closely associated to serious hepatocyte damage. On the contrary, the abnormal serum AST levels could be correlated to liver damage but also to numerous other body tissues. Lipid assessment in serum revealed a two-fold increase in total circulating cholesterol and a mild increase in triglycerides in ApoE^–/–^HFD compared to ApoE^–/–^ HFD + APH mice. Serum lipid analysis showed that consuming APH for 12 weeks produced significant cholesterol- and triacylglycerol-lowering effects, despite the high fat content of the diet. These findings agree with the results reported by other authors in previous studies that have demonstrated that fish-derived proteins or hydrolysates reduce body weight, fat mass and circulating levels of lipids. In particular, protein hydrolysates obtained from Atlantic herring, salmon and cod by-product wastes produced a significant hypocholesterolemic effect in obese Zucker rats [33]. Moreover, salmon fish protein hydrolysates showed a marked hypocholesterolemic and anti-atherogenic effect in ApoE knockout mice [36]. Decreased serum lipids are the most reported result of fish oil supplementation in humans [46], rats [47], and horse [48]. Although the exact mechanism responsible for this reduction has not yet been clarified, it has been suggested that fish oil supplementation could downregulate enzymes associated with triglyceride synthesis and/or increase lipoprotein lipase activity and fatty acid oxidation [49]. The liver content of free cholesterol, cholesteryls ester and triacylglycerol were increased in ApoE^–/–^mice fed HFD, as previously demonstrated in other studies [14,29]. Interestingly, liver of mice fed with fish protein hydrolysates showed a 58.47% decrease in triacylglycerol content but did not display a significant reduction in free cholesterol and cholesteryl ester content. Chemical characterization of anchovy visceral protein hydrolysates employed in the current study showed a high content of essential amino acids (EAA), that is, approximately 42% of the total amino acids [39]. Interestingly, 38% of the total amino acid content was represented by proline, leucine, alanine and aromatic acids [39]. It has been demonstrated that peptides containing these amino acids are effective against dangerous free radicals [37,50], and that the high content of proline could play an important role in cholesterol metabolism, thus reducing the risk of cardiovascular diseases, such as atherosclerosis [37,50]. It has been reported that hepatic steatosis and liver injury associated with steatohepatitis in mice can be alleviated by branched-chain amino acid diet supplementation, and by suppressing gene expression and the protein level of fatty acid synthase (FAS) [51]. The general control nonderepressible 2 (GCN2) kinase activates the amino acid response signal transduction cascade. It has been reported that during prolonged leucine deprivation resulting in severe liver steatosis, lipid synthesis was upregulated in the livers of Gcn2^-/-^ mice due to failure to up-regulate peroxisome proliferator-activated receptor alpha (PPARα) expression [52]. PPARα is abundant in hepatocytes where it finely orchestrates the lipid metabolism regulating fatty acid degradation, synthesis, transport, storage and lipoprotein synthesis and degradation [52]. Pre-clinical studies have demonstrated that the restoration of dietary protein by supplementation with fish-derived protein increases hepatic expression of the two PPARα target genes (peroxisomal acyl-CoA oxidase 1 and mitochondrial carnitine palmitoyl-transferase-II) in mice previously fed with a protein-restricted diet [52]. This hepatoprotective action is most likely due to stimulation of mitochondrial and peroxisomal β-oxidation of fatty acids [52]. Altogether, the high content and composition of amino acids highlight the nutritional value of fish by-product hydrolysates, and since in addition to viscera hydrolysates, muscle, head and skin protein hydrolysates have also been proven to contain the same content of all the essential and non-essential amino acids, the potential of these products is worthy of investigation. Nowadays, fish protein hydrolysates are produced from several protein-rich fish by-product wastes [34], and by virtue of their functional proprieties, they are largely employed to improve human health by providing essential nutrients and protection from several diseases.

## 5. Conclusions

The current study assesses the effect of the administration of a 10% (*w*/*w*) anchovy protein hydrolysates diet supplementation for 12 weeks on attenuated high-fat diet-induced MAFLD in apolipoprotein-E-deficient mice as the experimental animal model. The results suggest an anti-obesity effect together with an improvement in lipid metabolism, liver fat content, and a significant attenuation of diet-induced liver alterations and hepatocyte injury. By virtue of their nutritional value and biological proprieties, anchovy by-product protein hydrolysates could be employed as a useful nutritional strategy in metabolic associated fatty liver disease prevention and treatment in the near future. The possibility of obtaining and using bioactive molecules from fish by-product wastes as potential nutraceuticals for several pathological conditions is worthy of future investigations, and also represents a key factor in reducing fish industry-derived pollution.
